# Effects of Whole-Body and Lower-Body Cold-Water Immersion on Exercise-Induced Pain Score, Muscle Damage Indices, and Maximal Voluntary Isometric Contractions

**DOI:** 10.3390/jcm14103287

**Published:** 2025-05-08

**Authors:** Jinseok Lee, Jeheon Moon, Namsu Kim

**Affiliations:** 1 Department of Physical Education, Daegu National University of Education, Daegu 42411, Republic of Korea; js0420@dnue.ac.kr; 2 Department of Physical Education, Korea National University of Education, Cheongju 28173, Republic of Korea; jhmoon@knue.ac.kr; 3 Department of Bio & Medical Big-Data (Bk21 plus), Gyeongsang National University, Jinju 52828, Republic of Korea

**Keywords:** cold-water immersion, maximal voluntary isometric contraction, muscle damage index, muscle mechanical properties

## Abstract

**Background/Objectives**: The aim of this study was to assess the effects of cold-water immersion (CWI) post-eccentric muscle contraction exercise on skin temperature, pain score, maximum voluntary isometric contraction (MVIC), muscle damage, and muscle mechanical properties. **Methods**: Twenty-seven male participants (age 20.6 ± 0.6; body mass 69.4 ± 8.1; body fat % 13.7 ± 4.3) were divided into three treatments: whole-body CWI treatment group (*n* = 9), lower-body CWI treatment group (*n* = 9), and control treatment group (*n* = 9). **Results**: MVIC did not show a significant interaction effect between group and time but demonstrated a significant main effect for time (*p* = 0.001). The pain scale demonstrated a significant interaction effect between group and treatment (*p* = 0.049), in addition to significant main effects for both time and treatment (both *p* = 0.001). While blood creatine kinase (CK) concentration revealed no significant interaction effect between group and time, a significant main effect was observed for time (*p* = 0.001). Blood lactate dehydrogenase (LDH) concentration showed both a significant interaction effect between group and time (*p* = 0.02) and a significant main effect for time (*p* = 0.001). The tensiomyography (TMG) results for Dm showed a significant interaction effect between group and treatment (*p* = 0.047), as well as a significant main effect for time (*p* = 0.001). **Conclusions**: Lower-body CWI is effective in reducing pain indices and blood LDH levels, a marker of muscle damage. It may serve as an effective method for preventing and minimizing pain and muscle damage, comparable to whole-body CWI.

## 1. Introduction

In the field of sports, various initiatives are designed to enhance the performance of elite athletes. To achieve these goals, training and periodization strategies are employed to maximize the effectiveness of the training regimen. However, overtraining can result in a decline in physical condition, an increased risk of injuries, elevated fatigue levels, and muscle damage. Hence, it is important to ensure adequate recovery following exercise or training [1].

Although recovery methods in sports vary depending on the event, active recovery (e.g., jogging and cycling) is commonly implemented [2]. Low-intensity running, which involves approximately 30–40% of maximum oxygen intake following high-intensity training, has been shown to reduce blood lactate concentration more rapidly than static rest [3]. Additionally, low-intensity running enhances lactate oxidation in skeletal muscles post-exercise, converting lactate into glucose or amino acids, thereby aiding in its removal [4,5]. However, it is important to note that this method has limitations in preventing muscle damage and maintaining exercise performance, both of which are critical for sustaining conditioning.

CWI has gained attention as an effective method for mitigating muscle damage and maintaining conditioning in athletes [6]. Research has indicated that CWI can reduce muscle soreness and biochemical indicators of muscle damage, including blood CK and LDH levels, and may affect muscle function in elite athletes after competition [7]. It also influences indicators of physical recovery, including heart rate variability and muscle power [8]. CWI involves immersing the body up to the iliac or clavicle in water at a temperature of 10–15 °C for 10 to 15 min and is widely used in sports for effective post-exercise recovery [9].

Previous studies have reported that whole-body immersion in cold water up to the clavicle after intense exercise attenuates the inflammatory response, reduces the increase in CK and LDH levels, elevates levels of the anti-inflammatory cytokine interleukin-10 (IL-10), and decreases levels of the pro-inflammatory cytokine interleukin-2 (IL-2) as well as interleukin-8 (IL-8), a chemokine involved in muscle damage recovery [10]. A study investigating the effects of lower-body CWI reported that applying CWI after exercise effectively reduced free oxygen radicals and blood lactate levels [11]. This method has been shown to lower the levels of CK and myoglobin, which are key indicators of muscle damage, more effectively than general sedentary recovery. Specifically, CWI reduces both the depth of tissue impact and temperature, thereby slowing nerve transmission speed, minimizing pain, and increasing hydrostatic pressure. It also reduces swelling and mass formation by decreasing cell membrane permeability and attenuates the immune response to tissue damage [12,13]. Further research has demonstrated that CWI effectively reduces LDH levels, alleviates muscle pain, and promotes muscle recovery [14]. Ultimately, post-exercise CWI effectively reduces the temperature of the exercised area, alleviates fatigue, decreases markers of muscle damage, and suppresses inflammatory responses. However, the effectiveness of CWI may vary depending on the depth of immersion. Several studies have primarily focused on the effects of CWI on the whole body and lower body, indicating that results tend to vary depending on the specific body parts submerged [15,16]. Christophe reported that CWI may be effective for intrinsic muscle factors, such as damage and recovery, but it is known to potentially have negative effects on muscle function [17]. Following high-intensity exercise, muscle inflammation occurs, resulting in elevated levels of CK and LDH [18]. CWI lowers tissue temperature, thereby attenuating the inflammatory response, and further reduces inflammation levels through vasoconstriction [19]. While there is a paucity of studies investigating the short-term effects on the improvement of CK and LDH levels, as well as on functional recovery, numerous studies have documented that CK and LDH contribute to inflammation, thereby negatively affecting both muscle function and recovery. Consequently, CK and LDH have been extensively utilized as representative biomarkers of muscle damage in existing research [20,21].

TMG has been recognized as an effective tool for addressing the limitations of earlier studies, particularly in observing muscle contraction characteristics and fatigue factors. In a study by [22], TMG was employed to examine changes in the muscle characteristics of soccer players who underwent four repetitions of 4 min CWI at 4 °C. The findings revealed that repeated CWI significantly altered muscle properties. Notably, the study found a significant reduction in maximal displacement (Dm) among participants subjected to the CWI protocol compared to a control group. Dm, a crucial parameter measured by TMG, indicates the peak displacement of muscle tissue during contraction and serves as a marker of muscle stiffness or muscle hypertrophy, which has been correlated with an increase in Dm, whereas severe muscle atrophy is associated with a decrease in Dm, suggesting that a reduction in Dm may serve as an indicator of increased muscle stiffness [23,24]. Therefore, the application of TMG in the assessment of muscle characteristics facilitates a more thorough evaluation of alterations in muscle function in response to varying depths of CWI. Moreover, MVIC serves as a critical parameter in the assessment of muscle function. Prior research indicates that CWI is acknowledged for its potential to reduce MVIC [25,26]. However, the specific effects of whole-body CWI versus lower-body CWI in the context of CWI have not been comprehensively explored. Consequently, there is a notable paucity of studies examining the changes in TMG metrics and MVIC, particularly in relation to muscle function following both whole-body CWI and lower-body CWI. Furthermore, although whole-body CWI is widely used as a recovery method, it presents certain limitations, including the physical discomfort associated with prolonged exposure to cold water and the substantial volume of water required for immersion. If lower-body CWI proves to be effective in alleviating muscle soreness, reducing muscle damage, and enhancing muscle function recovery, it may serve as a more practical and efficient alternative to whole-body CWI.

The present study seeks to fill the existing research gap through two primary objectives: First, it aims to investigate the recovery of muscle damage biomarkers, TMG, and MVIC following whole-body CWI and lower-body CWI after high-intensity eccentric exercise. Second, this study aims to contribute to a broader understanding that may subsequently inform research focused on elite athletic performance. If lower-body CWI proves to be as effective as whole-body CWI in ameliorating muscle damage and enhancing muscle function, it may offer insights into more efficient and convenient methodologies for immersion-based treatments.

## 2. Materials and Methods

### 2.1. Design and Setting

**Figure 1:**
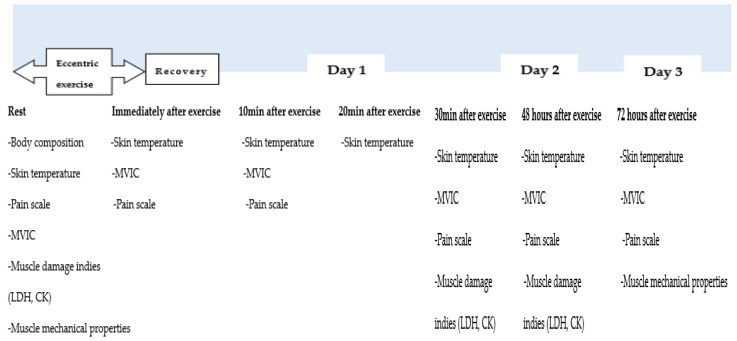
Procedure of experiment.

The procedure for conducting this study and the detailed steps of the experiment are illustrated in Figure 1.

This study was conducted at the G University Physical Education Science Research Institute in Gyeonggi Province, South Korea. Blood samples were collected from participants to assess changes in muscle damage indices across different treatment groups. In addition, participants underwent MVIC tests and TMG tests to investigate the mechanical muscle properties.

During their initial laboratory visit, participants underwent anthropometric assessments. They were instructed to refrain from eating and exercising for at least 12 h before the assessment. The participants wore only sportswear during the assessments, which included measurements of height, weight, and body fat percentage. These measurements were obtained using an automatic height scale (SD-102, Biospace Co., Seoul, Korea) and a bioelectrical impedance analysis (BIA) machine (Inbody 720, Biospace Co., Seoul, Korea). All participants performed eccentric exercises followed by their assigned treatment. Prior to the eccentric exercise, the baseline assessments included measurements of body composition, pain scale, skin temperature, MVIC, and mechanical muscle properties. Blood samples were collected from the main vein of the forearm (10 mL). The same parameters, except for body composition, were evaluated immediately after the eccentric exercise. Ten minutes post-exercise, skin temperature, pain scale, and MVIC were assessed, and 0.5 mL of blood was drawn from the finger. Skin temperature was assessed again twenty minutes post-exercise. At the thirty-minute mark post-exercise, skin temperature, MVIC, and mechanical muscle properties were reassessed, and blood samples were collected from the main vein of the forearm (10 mL). Further assessments were conducted forty-eight and seventy-two hours after the muscle contraction exercise, including skin temperature, pain scale, MVIC, and mechanical muscle properties, with blood collected from the main vein of the forearm (10 mL). Nutritional factors that could influence the results were meticulously controlled throughout the experiment, with participants required to maintain a detailed meal diary. Additionally, participants were educated and closely monitored to ensure external factors such as alcohol consumption, exercise, sleep, and smoking did not impact the research outcomes.

### 2.2. Participants

A total of thirty-three healthy adults were initially recruited and qualified for this study. The sample size was determined using G-power software 3.1.9.7 (https://g-power.software.informer.com/3.1/, 2 May 2023) based on the study’s design parameters. The sample size of thirty-three participants was determined based on the principles of the central limit theorem, which was considered in the calculation of the sample size for this study [27]. However, due to more than two absences or injuries during the assessment period, six individuals were excluded, resulting in a final cohort of twenty-seven participants: whole-body CWI treatment group (n = 9), age 20 ± 0 years, height 176 ± 6.4 cm, body mass 69.7 ± 9.0 kg, body fat percentage 14.0 ± 3.4%; lower-body CWI treatment group (n = 9), age 20 ± 2 years, height 174.9 ± 3.7 cm, body mass 68.8 ± 6.9 kg, body fat percentage 11.4 ± 3.8%; and control group (active recovery, n = 9), age 22 ± 3 years, height 174.6 ± 5.6 cm, body mass 69.8 ± 8.4 kg, body fat percentage 15.7 ± 5.6%. All participants received a thorough explanation of the study’s purpose, protocol, and associated risks before providing written informed consent. This study was approved by the Institutional Review Board of the Korea Institute of Sports Science, with the reference number (KISS-1806-032-01, 20 June 2018).

### 2.3. Skin Temperature

Skin temperature was assessed at rest; immediately after exercise; and at 10 min, 30 min, 48 h, and 72 h post-exercise. A skin temperature meter (62 MAX/62 MAX+ Users, FLUKE Co., Eindhoven, The Netherlands) was used to measure temperatures at the chest, upper arm, thigh, and calf. Each site was measured twice, and the higher value was used to calculate the average skin temperature using the formula [28]. Thermal imaging was also utilized to capture changes in body temperature, using a thermal imaging camera (T650SC, FLIR Co., Täby, Sweden) (Table 1).

### 2.4. MVIC

The MVIC of the knee joint was measured using an isokinetic dynamometer (Humac/Norm, Model 770, Computer Sports Medicine Inc., Stoughton, MA, USA). Muscle strength was assessed on the dominant-side femoral quadriceps muscle with the knee flexed at 60°, using an isometric contraction for 10 s to maintain maximum force [29], which corresponds to a zero-velocity (0°/s) condition. Assessments were conducted at rest; immediately after exercise; and at 30 min, 48 h, and 72 h post-exercise.

### 2.5. Pain Score

The pain score was developed based on previous studies, utilizing a 0 to 10 scale (0: no pain, 5: moderate pain, 10: maximum pain) [30]. Evaluations were conducted immediately after exercise and at 10 min, 30 min, 48 h, and 72 h post-exercise.

### 2.6. Muscle Damage Indices

Muscle damage indices were determined by analyzing blood LDH and CK concentrations. Blood samples were collected at rest; immediately after exercise; and at 30 min, 48 h, and 72 h post-exercise.

Blood samples were collected between 6:00 a.m. and 9:00 a.m. after the participants had fasted for at least 10 h. Upon arrival, the participants rested for 10–15 min before blood was drawn from the main vein of the forearm and the fingertip. LDH and CK analyses were performed on blood collected from the forearm vein, with serum processed at a specialized medical institution using a Cobas C702 analyzer (Roche Co., Munich, Germany). On the day of treatment, blood was collected at rest; immediately after eccentric exercise; and at 10, 20, and 30 min post-exercise. The blood samples for the LDH and CK analyses were collected from the main forearm vein (10 mL) at rest before eccentric exercise. With the exception of lactate, all blood analyses were performed at a specialized medical institution, and blood collection was conducted by a licensed nurse under the supervision of a specialist. The blood was stored in a sterile specimen container at room temperature for approximately 10 to 30 min for coagulation, after which the serum was obtained using a centrifuge (3000 RPM × 15T). The serum was then transferred to a specialized medical institution using a blood transport box maintained at 4 °C to 7 °C. Blood LDH and CK concentrations were analyzed using a Cobas C702 analyzer (Roche Co., Germany) on the same day.

### 2.7. Muscle Mechanical Properties

**Figure 2:**
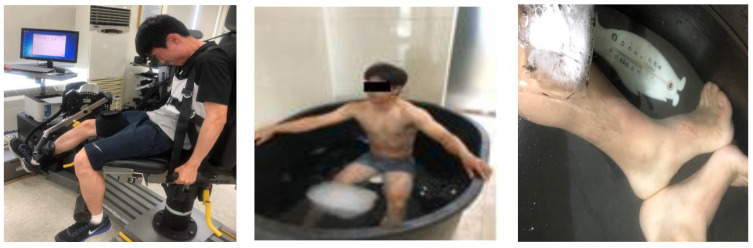
The eccentric exercise and CWI treatment.

Muscle mechanical properties were evaluated using TMG at four time points: at rest and at 30 min, 48 h, and 72 h post-exercise (GK40, Panoptik d.o.o., Ljubljana, Slovenia). The participants were positioned with their knee flexed at 120° for the assessment, with full knee extension defined as 180°. During the experiment, participants’ arms, legs, and torso were secured as illustrated in Figure 2. This angular reference is used consistently throughout this paper. Electrodes were placed at 5 cm intervals around the femoral muscle, with a sensor positioned at the center. Electrical stimulation commenced at 20 mA, increasing in 10 mA increments until peak muscle contraction was achieved. The parameters recorded included Dm, response time, and muscle relaxation time [31].

The TMG assessments focused on each participant’s stronger leg to minimize testing duration. The stronger leg was determined based on a questionnaire regarding the participant’s dominant leg and by referencing previous study [32]. Electrical stimulation started at 20 mA and was increased by 10 mA increments until the muscle’s Dm was reached. The response to each stimulus was recorded via a sensor connected to a computer, which calculated Dm, response time, and muscle relaxation time using specialized software. Dm is used to assess muscle tension or stiffness, where lower Dm values indicate increased muscle tension or stiffness [33]. Response time varies with the number of muscle fibers, being slower with a greater number of fibers and faster with fewer fibers [34]. Muscle relaxation time serves as an index for evaluating potential muscle nerve fatigue [25].

### 2.8. Eccentric Exercise Protocol

The participants warmed up by running on a treadmill for 5 min at 30–40% of their maximum heart rate (HRmax), followed by stretching of the femoral quadriceps and hamstrings for 5 min (20 s each). The eccentric exercise was performed using an isokinetic dynamometer (Humac/Norm, Model 770, Computer Sports Medicine Inc., USA). Seated with their torso and knees securely fastened, the participants executed knee joint flexion and extension at a velocity of 270°/s for 10 repetitions, which were characterized as concentric contractions. Following a 3 min rest interval, they engaged in eccentric exercises consisting of 10 sets of 10 repetitions at 90°/s, spanning from full extension 0° to 110°, with a 1 min rest between sets. This experimental design was implemented to elucidate the rationale behind conducting initial high-velocity concentric contractions before the eccentric exercise regimen [22].

### 2.9. Whole-Body and Lower-Body CWI, and Active Recovery Treatment Protocols

Post-exercise, the participants underwent whole-body or lower-body CWI treatments or active recovery. For CWI, the participants were submerged in water at 12 °C for 10 min, wearing only shorts (Figure 2). Whole-body CWI treatment involved submersion up to the collarbone level, while lower-body CWI treatment targeted the iliac region level. The control treatment engaged in active recovery, which involved running on a treadmill at 30–40% of HRmax for 10 min immediately following the eccentric exercise. Maximum heart rate was calculated by subtracting the participant’s age from 220. The participants assessed the water temperature using their hands and feet prior to immersion, and no restrictions were imposed during the immersion process. Additionally, there was no movement of the water during the CWI.

### 2.10. Statistical Analysis

The data for this study were analyzed using SPSS statistical software version 23.0 (IBM Corp., Armonk, NY, USA). Descriptive statistics, including means and standard deviations, were computed. To assess changes in body composition, skin temperature, MVIC, pain scale, muscle damage indices, and TMG in relation to recovery interventions and time points, a two-way repeated measure ANOVA was performed. Post hoc analyses were conducted using Bonferroni’s method. Furthermore, statistical analyses were performed using two-way repeated measure ANOVA to verify the effects of the treatment and time points, with post hoc comparisons conducted using the Bonferroni method. The statistical effect size was evaluated using eta squared (η^2^), with effect sizes interpreted as small (η^2^ ≈ 0.01), medium (η^2^ ≈ 0.06), and large (η^2^ ≥ 0.14), based on Cohen’s (1988) guidelines [35]. The statistical significance level was set at α = 0.05.

## 3. Results

### 3.1. Changes in Average Skin Temperature

Changes in skin temperature according to the recovery treatment method are presented in Table 2. The average skin temperature demonstrated a significant interaction effect between treatment and timing (*p* = 0.001). The post hoc test results indicate that, for the whole-body CWI treatment group, skin temperature was significantly lower at 10 min after exercise compared to at rest and immediately after exercise (both *p* < 0.05). Additionally, the average skin temperature was significantly lower at 10 min after exercise compared to 48 h and 72 h after exercise (both *p* < 0.05). In an investigation of the lower-body CWI treatment group, it was found that the average skin temperature 10 min after exercise, occurring immediately after the CWI, was significantly higher than at rest and immediately post-exercise (both *p* < 0.05). This observation suggests a potential rewarming effect following CWI, which may account for the elevated temperatures at the 10 min interval. Furthermore, skin temperature at this time point was significantly lower than at 30 min, 48 h, and 72 h post-exercise (*p* < 0.05 for each comparison). The effect sizes for these differences were calculated using partial eta-squared. Clarification of the protocol’s timing and procedures might be needed to address any potential misunderstandings regarding these findings. In the context of the control treatment group, the average skin temperature at 10 min post-exercise was significantly lower compared to at rest (*p* < 0.05) and was also significantly lower than at 30 min, 48 h, and 72 h post-exercise (*p* < 0.05 for each comparison). The observed increases in temperature at 48 and 72 h following passive recovery may be attributed to natural physiological processes such as delayed muscle rewarming or inflammation, which warrant further investigation to fully understand their impact. The whole-body CWI treatment group resulted in significantly lower skin temperatures than both the lower-body CWI and the control treatment group at 10 min after exercise (*p* < 0.05 for each comparison). When comparing the lower-body CWI treatment group, the whole-body CWI treatment group resulted in significantly lower average skin temperatures at 10 min after exercise (*p* < 0.05).

Chest temperature showed both a significant interaction effect between group and time (*p* = 0.001) and a significant main effect for time (*p* = 0.001). The post hoc test results indicate that, for the whole-body CWI treatment group, chest temperature was significantly lower at 10 min after exercise compared to 30 min, 48 h, and 72 h after exercise (all *p* < 0.05), and the for control treatment group, chest temperature was significantly lower at 10 min after exercise compared to 30 min, 48 h, and 72 h after exercise (all *p* < 0.05).

Thigh temperature showed both a significant interaction effect between group and time (*p* = 0.001) and a significant main effect for time (*p* = 0.001). The post hoc test results indicate that, for the whole-body CWI treatment group, thigh temperature was significantly lower at 10 min after exercise compared to 30 min, 48 h, and 72 h after exercise (all *p* < 0.05) and was significantly lower at 30 min after exercise compared to 72 h after exercise (*p* < 0.05). For the lower-body CWI treatment group, thigh temperature was significantly lower at 10 min after exercise compared to 30 min, 48 h, and 72 h after exercise (respectively, *p* < 0.05).

Upper arm temperature showed no significant interaction effect between group and time, but a significant main effect was observed for treatment (*p* = 0.001).

Calf temperature showed both a significant interaction effect between group and time (*p* = 0.001) and a significant main effect for time (*p* = 0.001). The post hoc test results indicate that, for the whole-body CWI treatment group, calf temperature was significantly lower at 10 min after exercise compared to 48 h and 72 h after exercise (both *p* < 0.05) and was significantly lower at 30 min after exercise compared to 48 h and 72 h after exercise (both *p* < 0.05). For the lower-body CWI treatment group, calf temperature was significantly lower at 10 min after exercise compared to 30 min and 48 h after exercise (both *p* < 0.05) and was significantly lower at 30 min after exercise compared to 48 h and 72 h after exercise (both *p* < 0.05). For the control treatment group, calf temperature was significantly lower at IAE compared to 48 h and 72 h after exercise (both *p* < 0.05).

### 3.2. Changes in MVIC

Table 3 presents the changes in MVIC according to the recovery treatment method. The MVIC showed a no significant interaction effect between group and time but demonstrated a significant main effect for time (*p* = 0.001).

### 3.3. Changes in Pain Scale

The changes in pain scale scores following the whole-body CWI treatment group, lower-body CWI treatment group, and control treatment group after eccentric muscle contraction exercise are presented in Table 4. The pain scale scores demonstrated a significant interaction effect between CWI treatment and time (*p* = 0.049), in addition to significant main effects for both time and treatment (both *p* < 0.05). The post hoc test revealed several significant differences across time points. The whole-body and low-body CWI treatment groups resulted in significantly lower pain scale scores than the control treatment group at 30 min and 48 h post-exercise (both *p* < 0.05).

### 3.4. Changes in Muscle Damage Indices

Changes in the blood muscle damage indices according to the recovery treatment method are depicted in Table 5. The blood CK concentration revealed no significant interaction effect between group and time, but a significant main effect was observed for time (*p* = 0.001). Lactate dehydrogenase (LDH) showed both a significant interaction effect between group and time (*p* = 0.02) and a significant main effect for time (*p* = 0.001). As a result of the post hoc test, the blood LDH concentration in the lower-body CWI treatment group was significantly lower at 30 min post-exercise compared to 72 h post-exercise (*p* < 0.05). When comparing the control treatment group, the blood LDH concentration at 48 h post-exercise was significantly lower in both the whole-body CWI treatment and lower-body CWI treatment compared to the active recovery treatment (both *p* < 0.05).

### 3.5. Changes in Muscle Mechanical Properties

The TMG changes according to the recovery treatment method are shown in Table 6. Dm demonstrated a significant interaction effect between treatment and time (*p* = 0.047), as well as a significant main effect for time (*p* = 0.001). Post hoc tests revealed significant differences between time points. For the whole-body CWI treatment group, Dm was significantly lower at 30 min post-exercise compared to at rest (*p* < 0.05) and significantly lower at 30 min after exercise compared to 48 h and 72 h after exercise (both *p* < 0.05). Similarly, for both the lower-body CWI and control treatment groups, Dm was significantly lower at 30 min after exercise compared to at rest (*p* < 0.05) and significantly lower at 30 min after exercise compared to 48 h after exercise (both *p* < 0.05). TC showed a significant main effect based on the time (*p* = 0.044), whereas Tr did not show any main effect.

## 4. Discussion

This study investigated the effects of whole-body and lower-body CWI, and active recovery (control treatment) treatments on average skin temperature, MVIC, muscle damage indices, and muscle mechanical properties in healthy adults following high-intensity eccentric exercise. We observed significant changes in average skin temperature following CWI. Both whole-body and lower-body CWI, as well as active recovery, exhibited statistically significant differences in average skin temperature across different time intervals. Our findings align with those of earlier studies. For instance, one study reported that CWI treatment significantly reduced skin temperature for high-intensity exercise compared to a control group [36]. Similarly, the study in [37] found that CWI treatment administered to 11 adult men following maximal aerobic exercise resulted in significant deviations in average skin temperature compared to a control group during a 20 min assessment period. These consistent findings suggest that employing CWI treatment post-training or competition may help mitigate inflammation and pain levels, thereby facilitating rapid fatigue recovery. Temperature fluctuations were categorized based on body regions as follows: chest temperature exhibited a significant interaction effect between the administered treatment and assessment timing, with the lowest temperature recorded 10 min post-exercise, indicating statistical significance. These findings demonstrated that the lowest upper-body temperature occurred 10 min after the CWI treatment following high-intensity cycle training, with statistical significance supporting the outcomes of this study [38]. This result aligns with the statistical significance observed in our study. Additionally, consistent with similar effects seen in low-temperature immersion treatments, the use of a cooling tube showed no significant change in temperature 30 min post-exercise [39]. This suggests that the whole-body CWI treatment may be preferable for more effective temperature reduction post-exercise. An analysis of prior research on CWI indicates that applying CWI exclusively to the exercised body parts following resistance training resulted in reduced muscle growth [40]. Therefore, in light of this finding, further investigation into the effects of exercise type and CWI protocols is warranted.

Generally, studies have shown that maintaining a water temperature of 8 °C during CWI treatment is effective in reducing body temperature [41]. Several studies have reported that 10 min of CWI treatment at temperatures ranging from 10 °C effectively reduces tissue temperature, blood circulation, capillary blood volume, and metabolic activity in skeletal muscle after exercise [42]. Research has demonstrated that following resistance exercise, CWI treatment from a sitting position to the collarbone resulted in a decrease in muscle temperature by approximately 7 °C compared to active recovery treatment [41]. They also observed a decrease in venous blood oxygen saturation immediately after treatment, lasting up to 2 h post-treatment, along with a reduction in blood Mb concentration at 2, 4, and 6 h post-treatment, indicating an advantage in relieving fatigue. Combining the results of these studies, it has been established that lower-body CWI effectively reduces lower-body and thigh temperatures. Furthermore, whole-body CWI treatment is effective in reducing average skin temperature by decreasing temperatures in the chest, lower body, and thighs.

Blood CK and LDH concentrations are well-established indicators of muscle damage [20,21]. In this study, a significant main effect of the treatment was observed in blood CK concentration, with no significant difference noted between the whole-body CWI treatment group, lower-body CWI treatment group, and active recovery treatment group (control treatment group). Notably, at 48 h post-exercise, the blood CK concentration following the whole-body CWI treatment group and lower-body CWI treatment group was lower than that following active recovery treatment, with whole-body CWI resulting in the lowest levels. Similarly, blood LDH concentration was significantly lower at 48 h post-exercise for both the whole-body CWI treatment group and lower-body CWI treatment group compared to active recovery treatment. In particular, the lowest blood LDH concentration was observed in the whole-body CWI treatment group results. Based on the statistical significance of blood LDH concentration, it can be concluded that CWI treatment had a positive effect on the muscle damage indices, regardless of the treatment site. However, the primary effect of the treatment was confirmed only for blood CK concentration, indicating no significant difference between the whole-body CWI treatment group and active recovery treatment group. Despite this, the numerical data showed that blood CK concentration was lower following both the whole-body and lower-body CWI treatments compared to active recovery treatment 48 h post-exercise, with whole-body CWI treatment yielding the lowest levels. Blood LDH concentration also followed a similar trend, being significantly lower 48 h post-exercise in the whole-body CWI treatment group and lower-body CWI treatment group compared to the active recovery treatment group. In particular, whole-body CWI treatment displayed the lowest numerical blood LDH concentration. Considering the statistical significance of blood LDH concentration, it can be concluded that CWI treatment had a positive effect on the muscle damage index, irrespective of the treatment site. Active recovery over an adequate duration may be more effective for muscle recovery than CWI when considering muscle damage indices. It is important to note that blood CK and LDH concentrations tend to be higher in the evening than in the morning [41]. To control for this variability, data collection in this study was conducted in the early morning during periods of low activity. Previous research has indicated that exercise can influence daily activity levels [43]. Although this study observed enhanced outcomes associated with CWI treatment, potential influences from daily activities were likely minimized, as all participants were assessed at the same time. Based on these findings, CWI treatment is an effective approach for mitigating indicators of muscle damage, regardless of the treatment site.

Post-exercise CWI treatment resulted in an increase in MVIC at 24 h and 48 h post-treatment. Additionally, there was a reduction in blood CK and Mb levels, suggesting that CWI treatment offers benefits for muscle damage recovery [44]. A study reported that 10 min of lower-body CWI at 10 °C decreased femoral and lower-body temperatures while enhancing MVIC and vertical jump capacity to steady-state levels after 24 h [45]. Conversely, some other research found no changes in isometric muscle strength in adult men following 20 min of CWI treatment or control treatment at water temperatures of 5 °C and 15 °C after inducing muscle fatigue through drop jumping. However, these studies reported faster recovery with the CWI treatment compared to the control treatment in recoil jumping [46]. Additionally, other studies indicated that CWI treatment at 10 ± 1 °C for 10 min did not lead to significant changes in MVIC, passive longitudinal stiffness, or passive transverse stiffness compared to the control treatment [26]. In this study, only the main effect of time was observed, indicating no significant difference in muscle strength recovery between whole-body CWI treatment, lower-body CWI treatment, and active recovery treatment. However, the MVIC at 48 h and 72 h post-exercise increased in the following order: whole-body CWI treatment, lower-body CWI treatment, and active recovery treatment. In other studies, it has been reported that MVIC temporarily decreases further with low-temperature immersion [17,47]. However, it is clear that while MVIC decreases temporarily depending on the duration or time, it commonly recovers after 24 or 48 h. Whole-body CWI and lower-body CWI do not seem to confer immediate benefits to muscle function when compared to dynamic recovery methods [24]. However, they are posited to more effectively enhance potential muscle function recovery by mitigating inflammation [48]. Despite these findings, the generalization of such results may be problematic. Depending on the timing, generalizing these findings presents challenges. Future studies should aim to clearly identify the effect of MVIC based on the low-temperature immersion area by recruiting a larger number of study participants and simplifying the treatment protocol.

Dm, the primary variable measured by TMG, represents the furthest travel distance of muscle movement caused by contraction, allowing for the assessment of muscle stiffness or tension. An increase in Dm indicates muscle expansion, while a decrease suggests muscle atrophy. Consequently, a decreasing Dm signifies increased muscle stiffness [26]. There are also cases where Dm increases with stable rest, leading to opposite results [27]. Additionally, Tc refers to the duration of muscle contraction, with longer Tc indicating greater muscle fatigue. Tr represents the muscle relaxation phase and serves as an index to evaluate potential muscle nerve fatigue. Research has reported that the application of CWI was reported to have a positive effect over time on leg muscle contraction characteristics, as assessed using TMG [49]. Significant changes associated with TMG have been documented, demonstrating its utility in various research contexts. As a result, in this study, a significant decrease in Dm was observed 30 min post-exercise across all treatment groups compared to baseline. When examining the MVIC results of this study, caution should be exercised in the use of CWI if immediate muscle strength is required after treatment. It is crucial to be aware that prolonged exposure to cooling can lead to burns and muscle contractions, necessitating cautious application [50]. In both the CWI and active recovery treatments, Dm decreased significantly 30 min post-exercise, indicating that both CWI and active recovery are effective in increasing muscle tension and stiffness. However, examining the numerical changes, Dm was significantly lower 30 min post-exercise in both the whole-body CWI and lower-body CWI treatments compared to active recovery. Additionally, only the main effect of the time point was observed in Tc, with no interaction effect or main effect of the treatment and time points observed in Tr. In a study by [51], participants performed a total of eight sets of recoil jumps for 30 s, followed by CWI treatment at 12 °C for 12 min. The study found no significant changes in the TMG variables of the vastus lateralis and the biceps femoris, and no statistical significance was observed in Tc and Tr between the treatment and time points. These results suggest the need to consider various muscle areas in future investigations. In this study, only TMG for the vastus lateralis was examined to assess muscle characteristics following CWI treatment after eccentric exercise. However, for more comprehensive verification, investigating TMG for various muscle areas, including the outer and inner thigh muscles, is essential. Additionally, due to the potential limitations posed by the relatively small sample size or extensive assessment points, observing changes in TMG across various muscles is crucial to minimize the impact of these factors in future research.

### Methodological Quality and Limitation

This study demonstrated that lower-body CWI is comparably effective to whole-body CWI in facilitating recovery from muscle damage and indicates that lower-body CWI may serve as a practical and accessible alternative for implementation. While the results indicate the potential of lower-body CWI as a recovery intervention for muscle damage in adult males, caution is advised when generalizing these findings to other populations, such as females, older adults, and adolescents, who require additional validation studies. This is because the study involved twenty-seven physically healthy adult male participants, with only nine participants assigned to each treatment condition. Due to logistical challenges associated with frequent blood sampling and repeated measurements, it was impractical for participants to undergo all three treatment protocols. Consequently, they were assigned to different treatment groups, presenting a methodological limitation in assessing CWI effects within the same individuals. The short duration further limits the findings. Future research should extend this study over a longer period and include these variables to enhance reliability and validity. Further research is required to substantiate these effects. To improve the generalizability and robustness of these findings, future research should involve larger sample sizes and adopt repeated-measures designs using the same participants to more accurately assess the effects of CWI on pain scores, muscle damage indices, and MVIC. Furthermore, previous research has identified sufficient sleep, proper nutrition, and adequate hydration as critical determinants impacting the efficacy of CWI [52]. It is posited that a thorough assessment of variables such as the subject’s physiological status, circadian rhythm, and training periodization, conducted prior to the administration of CWI, may enhance its overall effectiveness.

## 5. Conclusions

The results of this study indicate that whole-body and lower-body CWI treatments both effectively reduce thigh and calf temperatures, with whole-body CWI treatment showing particular efficacy in lowering average skin temperature by decreasing chest, calf, and thigh temperatures. Additionally, both the whole-body and lower-body CWI treatments significantly decreased the pain score 30 min and 48 h post-exercise compared to active recovery, confirming the pain relief benefits of CWI treatments.

Regarding MVIC, significant differences were observed at every time point for the whole-body CWI treatment, lower-body CWI treatment, and active recovery treatment, demonstrating that all three treatment approaches improve MVIC. Compared to the active recovery treatment, the whole-body and lower-body CWI treatments significantly reduced blood LDH concentration at 48 h post-exercise, suggesting that both CWI treatments effectively mitigate muscle damage indicators.

As for Dm, the whole-body CWI treatment, lower-body CWI treatment, and active recovery treatment significantly decreased Dm 30 min post-exercise compared to at rest. Moreover, for the CWI treatments, Dm was significantly lower at 30 min post-exercise compared to 48 h post-exercise.

In conclusion, both the whole-body CWI and lower-body CWI treatments have been observed to effectively reduce pain indices and blood LDH levels. Based on these findings, it is proposed that lower-body CWI may offer a more convenient approach compared to whole-body CWI for the efficient application of CWI.

## Figures and Tables

**Table 1:** Formula for calculating average skin temperature.

**Average skin temperature = 0.3 × (Chest + Upper arm) + 0.2 × (Thigh + Calf** **)**

**Table 2:** Changes in skin temperature according to recovery treatment method.

Variables	Treatment Group	Rest	IAE	10 min After Exercise	30 min After Exercise	48 h After Exercise	72 h After Exercise	Sig	η^2^
Average skin temperature (°C)	Whole-body CWI (n = 9)	33.20 ± 0.66 ^c,d^	32.72 ± 0.70 ^c,d^	28.63 ± 1.00 ^#,†,e,f^	31.61 ± 0.49 ^†^	33.23 ± 0.49	33.10 ± 0.82	Treatment 0.761	0.022
	Lower-body CWI (n = 9)	32.55 ± 0.50 ^c^	32.32 ± 1.02 ^c^	30.84 ± 1.39 ^d,e,f^	32.33 ± 0.93	32.57 ± 0.94	33.04 ± 0.69	Time 0.001 *	0.742
	Control (n = 9)	32.40 ± 0.78 ^c^	31.81 ± 0.62	31.14 ± 0.91 ^d,e,f^	32.35 ± 0.79	32.35 ± 0.79	32.76 ± 0.65	Treatment × Time 0.001 *	0.488
Chest temperature (°C)	Whole-body CWI (n = 9)	33.58 ± 1.03 ^c^	32.29 ± 0.99 ^c^	26.41 ± 2.26 ^#,†,d,e,f^	31.61 ± 1.38 ^#,†^	33.72 ± 0.83	33.44 ± 1.72	Treatment 0.111	0.167
	Lower-body CWI (n = 9)	33.17 ± 1.16	31.61 ± 1.18	31.90 ± 1.40	33.23 ± 1.58	32.84 ± 1.27	33.19 ± 1.36	Time 0.001 *	0.595
	Control (n = 9)	32.68 ± 1.26 ^c^	31.40 ± 0.97	30.41 ± 1.02 ^d,e,f^	32.77 ± 1.71	33.44 ± 1.58	33.11 ± 1.74	Treatment × Time 0.001 *	0.441
Thigh temperature (°C)	Whole-body CWI (n = 9)	32.64 ± 0.82 ^c^	32.97 ± 1.51 ^c,d^	26.90 ± 1.92 ^†,d,e,f^	31.12 ± 1.20 ^f^	32.94 ± 0.73	32.86 ± 0.50	Treatment 0.423	0.069
	Lower-body CWI (n = 9)	32.33 ± 1.22 ^c^	33.30 ± 1.51 ^c^	29.17 ± 2.47 ^d,e,f^	31.79 ± 1.03	32.22 ± 1.18	32.77 ± 0.81	Time 0.001 *	0.691
	Control (n = 9)	31.93 ± 0.97	32.56 ± 1.16	31.27 ± 2.68	32.30 ± 1.16	32.37 ± 0.87	32.77 ± 0.71	Treatment × Time 0.001*	0.422
Upper arm temperature (°C)	Whole-body CWI (n = 9)	33.67 ± 0.50	33.43 ± 0.80	33.22 ± 1.07	32.92 ± 0.96	33.58 ± 0.75	33.18 ± 0.91	Treatment 0.036 *	0.242
	Lower-body CWI (n = 9)	32.42 ± 0.61	32.87 ± 1.28	32.87 ± 1.79	33.18 ± 1.08	32.78 ± 0.85	32.89 ± 0.84	Time 0.399	0.041
	Control (n = 9)	32.86 ± 1.10	32.44 ± 0.55	31.83 ± 0.80	32.66 ± 0.67	32.88 ± 0.84	32.79 ± 0.69	Treatment × Time 0.128	0.121
Calf temperature (°C)	Whole-body CWI (n = 9)	32.55 ± 1.12 ^c,d^	32.23 ± 0.95 ^c,d^	26.63 ± 1.41 ^†,e,f^	30.41 ± 0.67 ^#,e,f^	32.18 ± 0.80	32.79 ± 0.58	Treatment 0.206	0.140
	Lower-body CWI (n = 9)	32.01 ± 0.66 ^c^	31.39 ± 0.46 ^c^	28.00 ± 2.30 ^#,d,e^	30.18 ± 0.58 ^#,e,f^	32.13 ± 1.47	32.79 ± 0.66	Time 0.001 *	0.755
	Control (n = 9)	31.79 ± 0.75	30.83 ± 1.08 ^e,f^	31.11 ± 0.88	31.39 ± 0.94	32.15 ± 0.52	32.26 ± 0.80	Treatment × Time 0.001 *	0.529

Values are mean ± SD, *: *p* < 0.05., CWI: cold-water immersion, IAE: immediately after exercise, ^#^: differences compared to the lower-body CWI treatment group, ^†^: differences compared to the control treatment group, ^c^: differences 10 min post-exercise, ^d^: differences 30 min post-exercise, ^e^: differences 48 h post-exercise, ^f^: differences 72 h post-exercise.

**Table 3:** Changes in MVIC according to recovery treatment method (%BW).

Variables	Treatment Group	Rest	IAE	30 min After Exercise	48 h After Exercise	72 h After Exercise	Sig	η^2^
MVIC (%BW)	Whole-body CWI (n = 9)	353.56 ± 52.99	306.22 ± 57.14	327.11 ± 57.71	346.44 ± 37.55	357.89 ± 44.90	Treatment 0.131	0.156
	Lower-body CWI (n = 9)	342.89 ± 30.25	274.78 ± 39.77	319.56 ± 48.39	330.33 ± 27.35	344.67 ± 46.24	Time 0.001 *	0.466
	Control (n = 9)	323.56 ± 48.27	265.89 ± 51.57	277.89 ± 65.67	283.00 ± 66.67	316.89 ± 78.69	Treatment × Time 0.452	0.075

Values are mean ± SD, *: *p* < 0.05, CWI: cold-water immersion, IAE: immediately after exercise, BW: body weight.

**Table 4:** Changes in pain score according to recovery treatment method.

Variables	Treatment Group	Rest	IAE	30 min After Exercise	48 h After Exercise	72 h After Exercise	Sig	η^2^
Pain scale (score)	Whole-body CWI (n = 9)	0.67 ± 1.12 ^b,c^	7.11 ± 2.14 ^c,d,e^	2.89 ± 1.36 ^†^	1.11 ± 1.53 ^†^	1.22 ± 1.20	Treatment 0.001 *	0.486
	Lower-body CWI (n = 9)	0.78 ± 1.30 ^b,c^	6.33 ± 1.50 ^c,d,e^	2.56 ± 1.01 ^†,e^	1.00 ± 1.23 ^†^	0.44 ± 0.53	Time 0.001 *	0.826
	Control (n = 9)	0.89 ± 0.78 ^b,c,d^	8.00 ± 1.11 ^c,d,e^	4.44 ± 1.42 ^e^	3.78 ± 1.64 ^e^	1.22 ± 1.09	Treatment × Time 0.049 *	0.156

Values are mean ± SD, *: *p* < 0.05., CWI: cold-water immersion, IAE: immediately after exercise; ^†^: differences compared to the control treatment group, ^b^: differences immediately post-exercise, ^c^: differences 10 min post-exercise, ^d^: differences 30 min post-exercise, ^e^: differences 48 h post-exercise, differences 72 h post-exercise.

**Table 5:** Changes in muscle damage indices according to recovery treatment method.

Variables	Treatment Group	Rest	IAE	30 min After Exercise	48 h After Exercise	72 h After Exercise	Sig	η^2^
CK (IU/L)	Whole-body CWI (n = 9)	207.56 ± 77.40	230.00 ± 78.34	240.89 ± 77.28	311.33 ± 160.75	235.44 ± 88.94	Treatment 0.714	0.028
	Lower-body CWI (n = 9)	209.22 ± 89.34	251.33 ± 106.12	241.56 ± 106.14	360.44 ± 168.68	308.11 ± 183.98	Time 0.001 *	0.400
	Control (n = 9)	175.00 ± 74.05	189.44 ± 41.36	196.56 ± 52.85	391.11 ± 184.16	261.11 ± 109.28	Treatment × Time 0.316	0.092
LDH (U/L)	Whole-body CWI (n = 9)	249.63 ± 40.67	275.25 ± 52.01 ^d,e^	232.50 ± 38.70	204.88 ± 46.55 ^†^	198.13 ± 47.35	Treatment 0.759	0.023
	Lower-body CWI (n = 9)	224.88 ± 41.52	241.25 ± 48.44	234.00 ± 47.85 ^e^	219.00 ± 61.96 ^†^	199.63 ± 33.76	Time 0.001 *	0.378
	Control (n = 9)	225.25 ± 37.09	268.13 ± 61.46	254.13 ± 30.69	268.00 ± 40.08	214.13 ± 44.91	Treatment × Time 0.027 *	0.171

Values are mean ± SD, *: *p* < 0.05, CWI: cold-water immersion, IAE: immediately after exercise; ^†^: differences compared to the control treatment group, ^d^: differences 48 h post-exercise, ^e^: differences 72 h post-exercise.

**Table 6:** Changes in TMG according to recovery treatment method.

Variables	Treatment Group	Rest	30 min After Exercise	48 h After Exercise	72 h After Exercise	Sig	η^2^
Dm (mm)	Whole-body CWI (n = 9)	7.3 ± 1.8 ^b^	4.5 ± 2.2 ^c,d^	7.6 ± 2.4	7.5 ± 1.8	Treatment 0.839	0.014
	Lower-body CWI (n = 9)	7.3 ± 1.4 ^b^	4.2 ± 2.2 ^c^	7.3 ± 2.4	6.4 ± 2.8	Time 0.001 *	0.485
	Control (n = 9)	7.1 ± 2.5 ^b^	6.1 ± 2.2 ^c^	7.3 ± 2.3	6.6 ± 1.6	Treatment × Time 0.047 *	0.165
Tc (ms)	Whole-body CWI (n = 9)	29.9 ± 6.0	32.1 ± 9.0	29.0 ± 5.4	29.7 ± 4.3	Treatment 0.373	0.079
	Lower-body CWI (n = 9)	27.9 ± 3.6	28.6 ± 6.2	27.6 ± 3.8	25.6 ± 5.1	Time 0.044 *	0.125
	Control (n = 9)	28.8 ± 3.3	31.6 ± 5.3	27.0 ± 5.2	28.2 ± 3.8	Treatment × Time 0.749	0.037
Tr (ms)	Whole-body CWI (n = 9)	34.7 ± 40.7	47.5 ± 48.6	33.1 ± 40.8	52.9 ± 72.2	Treatment 0.236	0.113
	Lower-body CWI (n = 9)	13.7 ± 4.1	22.3 ± 12.7	26.8 ± 27.1	19.1 ± 21.2	Time 0.194	0.066
	Control (n = 9)	23.3 ± 29.8	57.3 ± 45.4	26.8 ± 27.2	28.2 ± 35.0	Treatment × Time 0.498	0.067

Values are mean ± SD, *: *p* < 0.05, IAE: immediately after exercise, ^b^: differences 30 min post-exercise, ^c^: differences 48 h post-exercise, ^d^: differences 72 h post-exercise.

## Data Availability

The data are available on request due to privacy/ethical restrictions.

## Abbreviations

The following abbreviations are used in this manuscript:

CWI	Cold-Water Immersion
MVIC	Maximum Voluntary Isometric Contraction
CK	Creatine Kinase
LDH	Lactate DeHydrogenase
TMG	Tensiomyography
IL-10	Interleukin-10
IL-2	Interleukin-2
IL-8	Interleukin-8
Dm	Muscle’s Maximum Travel Distance
HRmax	Maximum Heart Rate
TC	Contraction Time
TR	Relaxation time
